# Multifocal ERG wavelet packet decomposition applied to glaucoma diagnosis

**DOI:** 10.1186/1475-925X-10-37

**Published:** 2011-05-17

**Authors:** Juan M Miguel-Jiménez, Sergio Ortega, Luciano Boquete, José M Rodríguez-Ascariz, Román Blanco

**Affiliations:** 1 Department of Electronics, University of Alcalá, 28701 Alcalá de Henares, Spain; 2 Department of Surgery, University of Alcalá, 28701 Alcalá de Henares, Spain

## Abstract

**Background:**

Glaucoma is the second-leading cause of blindness worldwide and early diagnosis is essential to its treatment. Current clinical methods based on multifocal electroretinography (mfERG) essentially involve measurement of amplitudes and latencies and assume standard signal morphology. This paper presents a new method based on wavelet packet analysis of global-flash multifocal electroretinogram signals.

**Methods:**

This study comprised twenty-five patients diagnosed with OAG and twenty-five control subjects. Their mfERG recordings data were used to develop the algorithm method based on wavelet packet analysis. By reconstructing the third wavelet packet contained in the fourth decomposition level (ADAA4) of the mfERG recording, it is possible to obtain a signal from which to extract a marker in the 60-80 ms time interval.

**Results:**

The marker found comprises oscillatory potentials with a negative-slope basal line in the case of glaucomatous recordings and a positive-slope basal line in the case of normal signals. Application of the optimal threshold calculated in the validation cases showed that the technique proposed achieved a sensitivity of 0.81 and validation specificity of 0.73.

**Conclusions:**

This new method based on mfERG analysis may be reliable enough to detect functional deficits that are not apparent using current automated perimetry tests. As new stimulation and analysis protocols develop, mfERG has the potential to become a useful tool in early detection of glaucoma-related functional deficits.

## Background

Alternative approaches using objective measures of glaucomatous neuropathy that do not rely on psycho-physiological or structural testing have been investigated in recent years. One approach has been to use electroretinography (ERG) to measure the changes in electrical activity generated by retinal ganglion cell bodies or axons in glaucoma [1].

Use of ERG to detect glaucoma requires isolation of specific components related to ganglion cell responses. Several ERG techniques involving measurement of light-adapted (photopic) and dark-adapted (scotopic) full-field flash electroretinograms have been investigated. This research into use of ERG in experimental glaucoma has produced clear evidence to suggest that electro-physiological tools can detect early functional changes in glaucoma [2].

A potentially more effective procedure is multifocal ERG (mfERG) [3], which takes simultaneous recordings of focal responses from over 100 different retinal regions and uses them to produce topographic representations of retinal response components.

The most common methods used to analyse the mfERG signal are based on amplitude and latency waveform analysis. For example, in subjects with primary open-angle glaucoma OAG, the amplitudes decrease while the latencies may increase [4]. Other approaches have used structural pattern analysis [5] to extract waveform identity patterns that may then be classified using a neural network. Zhou *et al. *have used the matching pursuit analysis method, a time-frequency analysis, to identify and characterize oscillatory potentials in the mfERG recording in primates [6].

The current paper represents a continuation of our previously published work [7].

The patients, methods of analysis and the results are new. Both studies have the same goal (glaucoma detection), but use different analysis tools: Discrete Wavelet Transform (DWT) in [7], versus Discrete Wavelet Packet Transform (DWPT) in this work. DWPT is an extension of the DWT to the full binary tree [8]. In the discrete wavelet packet transform, both the scaling and wavelet coefficients are subject to the high-pass and low-pass filtering when computing the next layer scaling and wavelet coefficients. DWPT permits the detail functions to be further split into two or more subbands [9], which offers a richer signal analysis (discontinuity in higher derivatives, self-similarity, etc.) [10].

The markers obtained in both works are clearly different. In the previous work we obtained a marker based on the latency of a valley and another marker based on the latency of an edge. In this paper we obtain a marker based on the slope of the baseline of some oscillatory potentials.

This paper studies application of the wavelet packet transform in mfERG analysis. By reconstructing the third wavelet packet contained in the fourth decomposition level (ADAA4) of the mfERG recording, it is possible to obtain a signal from which to extract a marker in the 60-80 ms time interval. This marker comprises oscillatory potentials with a negative-slope basal line in the case of glaucomatous recordings and a positive-slope basal line in the case of normal recordings and it can be reliably used to differentiate between normal and glaucomatous mfERG waveform signals.

## Methods

### Subjects

This study comprised twenty-five patients diagnosed with OAG and twenty-five control subjects (mean age: 47.5 (SD +/-2.5) for control group, 50.73 (SD +/- 3.8) for glaucoma group). For the purposes of analysis, normal and abnormal waveform databases were created from control subjects' and patients' mfERG recordings. These data were used to develop the algorithm method based on wavelet packet analysis.

Abnormal mfERG signals from glaucomatous patients were selected based on the same criteria followed in [7]. Informed consent was obtained from all participants. The University of Alcalá approved all the protocols and the study was conducted in accordance with the tenets of the Declaration of Helsinki.

Control subjects with normal eyes were included in this study to establish an age-matched norm. All control subjects' eyes had an intraocular pressure (IOP) of 21 mmHg or less (with no history of increased intraocular pressure). Control subjects were screened by means of an ophthalmoscopic examination to confirm the healthy appearance of the optic disc and all had normal Humphrey Visual Field (HVF) test results. All patients' eyes IOP were kept under 21 mmHg with glaucoma eye drops.

### mfERG recordings

**Figure 1:**
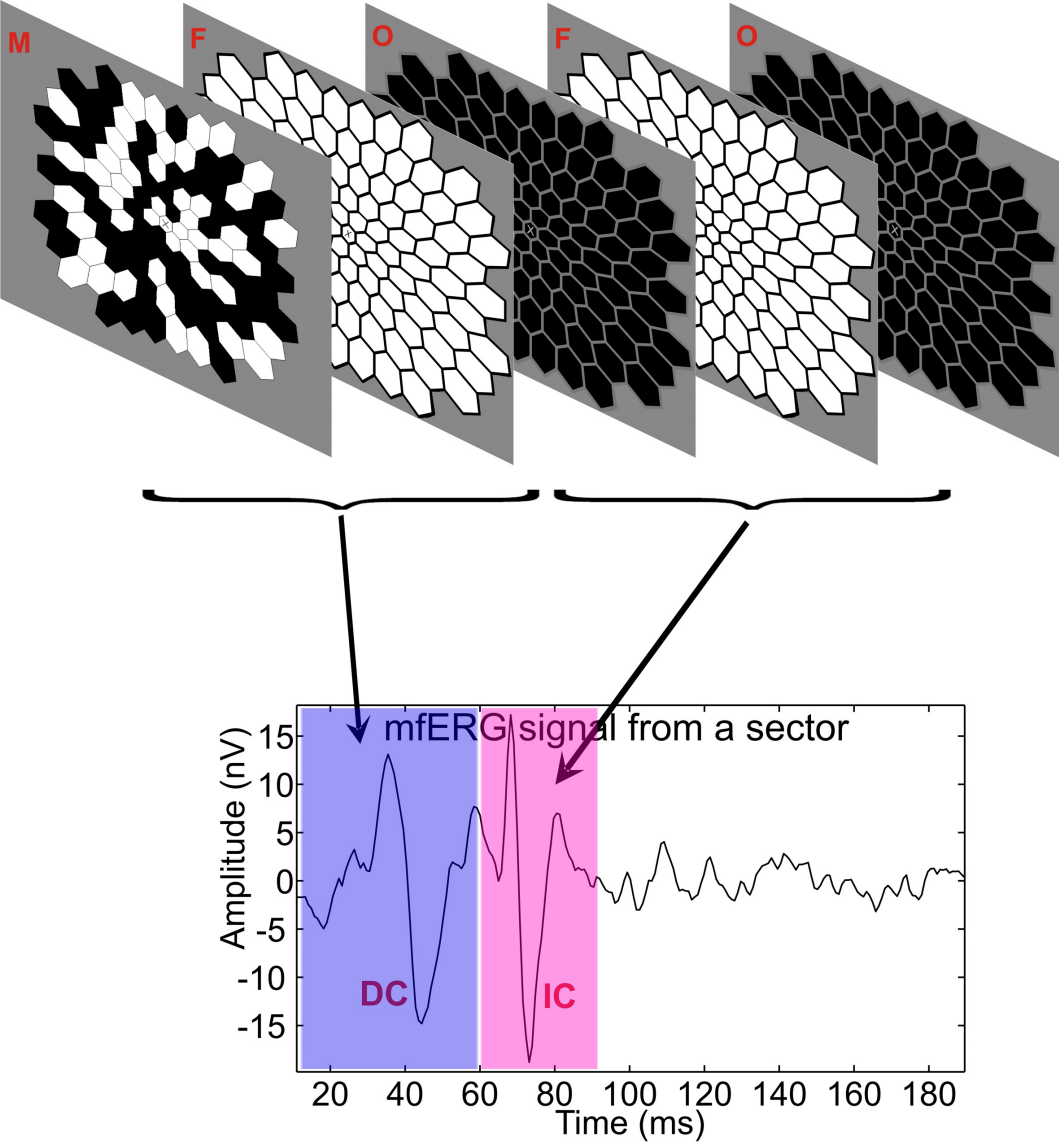
**Global-flash ERG multifocal stimulation**.

All patient recordings were taken using a commercially available multifocal system (VERIS System 5.1, Electro-Diagnostic Imaging, Inc., San Mateo, CA). The stimulus (Figure 1) consisted of an array of 103 densely packed hexagons tiling the central region of the visual field and about 45 degrees in diameter. The hexagonal stimulus elements were eccentrically scaled to equalize, approximately, the response amplitudes across the stimulated field. The stimulus array was presented on a 21-inch monochrome CRT monitor (NEC-FE2111SB) at a video frame rate of 75 Hz. The viewing distance for mfERG measurement was 32 cm. Each step of the ganglion cell response-enhancing stimulation protocol (M-F-O-F-O) consisted of five video frames. In the first frame (M), each stimulus hexagon was either independently flashed (200 cd/m^2^) or remained dark (<1.5 cd/m^2^) according to a pseudo-random binary m-sequence. After each multifocal stimulus frame (m-frame), the entire stimulus area flashed brightly (F) (100 cd/m^2^). The entire stimulus area then remained dark (O) for the next video frame, flashed brightly (F) for another frame and then was dark (O) again in the fifth frame. The sequence was then repeated, beginning with another m-frame of pseudo-random local stimulation followed by a full-field flash frame, a dark frame, a flash frame, a dark frame and so forth. The recordings were taken in ordinary indoor light conditions (room luminance: 100 cd/m^2^) with the pupil maximally dilated.

The stimulus was viewed through pupils (minimum diameter of 7 mm) pharmacologically dilated with tropicamide (1%). A Burian-Allen bipolar contact lens electrode (Hansen Ophthalmics, Iowa City, IA) was placed on the eye after administering a topical anaesthetic (0.5% proparacaine). Residual spherical refractive error was corrected with the VERIS™ refractor/camera unit mounted on the stimulating monitor. Fixation stability and alignment of the patient's pupil with the refractor's optics were monitored with a built-in infrared camera. Each monocular recording lasted approximately 9 minutes (m-sequence exponent m = 13). For patient comfort, the recording was taken in 16 segments of about 30 seconds each. Segments contaminated by eye movements were discarded and rerecorded. Signals were amplified with a Grass Neurodata Model 12 amplifier system (Grass Telefactor, NH) with a gain of 50,000, band-pass filters (10-300 Hz) and a sampling interval of 0.83 ms (1200 Hz).

Data were analysed off-line using the VERIS™ Science 5.1 software. Artefacts due to blinks and eye movements were removed from the data using two iterations of the VERIS™ artefact removal algorithm. One iteration of spatial filtering (averaging each focal waveform with 30% of its six neighbours) was applied to increase the signal-to-noise ratio. The response epoch selected for the analysis comprised the induced component (60-90 ms) judged to have the largest contribution from the optic nerve head component (ONHC). ONHC decomposition analysis and waveform quality assessment were performed using the VERIS™ 5.1 pro software. Further wavelet signal analysis was performed in MATLAB (MathWorks Inc, Natick, MA).

**Figure 2:**
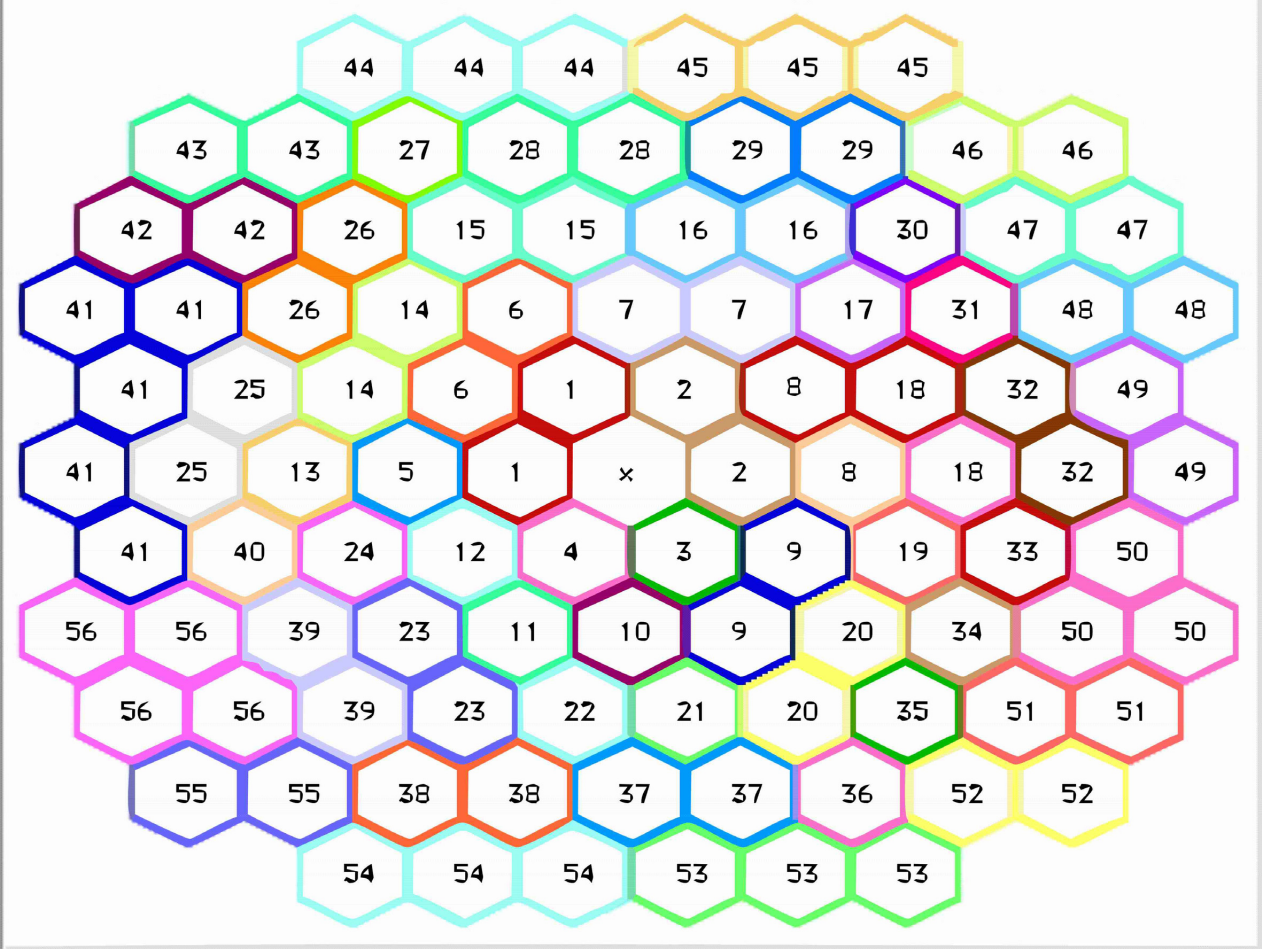
**Geometry of the multifocal stimulus and regrouping of the hexagons**.

Figure 2 shows the spatial distribution of mfERG sectors for the left eye. This distribution was obtained by regrouping and averaging the 103 hexagons to create a new 56-sector map to simplify analysis and improve the signal-to-noise ratio. The distribution for the right eye is a horizontal reflection of the left eye pattern. The 56-sector topography chosen is similar to that studied in automated perimetry, the clinical gold standard for visual field assessment.

Only glaucomatous sectors from patients affected by glaucoma made up the abnormal database, the total number of abnormal sectors was 723. Recordings from different numbers of patients could contribute to each sector. Sectors 1 and 2 had the least number of contributing records (3 each), sector 20 had the highest number of records (24) (SD = 5.33). The normal database was made up of 1400 sectors (25 controls, one eye per control, 56 sectors per eye). The minimum and maximum number of sectors per patient was 14 and 37, respectively (SD = 7.6).

### mfERG wavelet packet decomposition analysis

Multifocal ERG signals, like other biomedical signals, are subject to changes in frequency content over time. Wavelet transforms offer a strong alternative to Fourier methods in many medical applications because of their suitability for analysis of non-stationary signals. Wavelet analysis uses finite-length, oscillating, zero-mean waveforms, which tend to be irregular and asymmetrical. These are the windowing functions called mother wavelets. Wavelets can be expressed by two principal dimensions -- scaling and translation. A family of wavelets can be generated by a mother wavelet, Ψ(t), and these two dimensions according to the expression below:(1)

Thus, the values for scale *j *compress or stretch the wavelet. When stretched, the wavelet covers a larger time scale and is able to follow slower changes in the signal (related to low frequencies). When compressed, the wavelet captures finer signal details (related to high frequencies). The parameter *k *produces a translation of the wavelet in the time domain. The discrete wavelet transform [11-13] analyses the signal at different frequencies with different resolutions, using regions with windowing of different sizes and obtaining a two-dimensional time-frequency function as a result.

**Figure 3:**
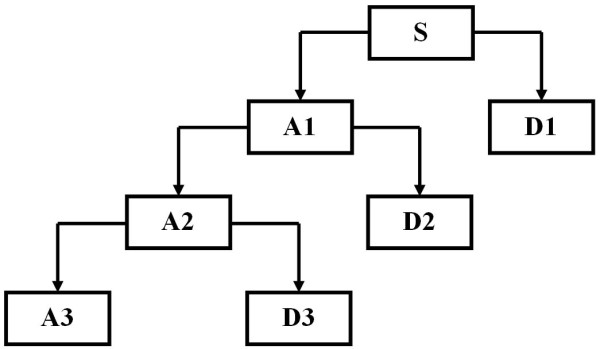
**Example of a three-level DWT**.

The DWT uses two function sets -- the scaling set, Φ_j,k_(t), and the wavelet set, Ψ_j,k_(t) -- associated with a low-pass and high-pass filter, respectively. The scaling function, Φ(t), defines the scales at which the wavelet operates. Detailed mathematical proofs and descriptions are found in Daubechies, Kaiser and Mallat [11-13]. At the first level of decomposition, the signal is divided into two parts: approximation (A_1_) and details (D_1_). Approximation of the signal provides its general morphological or low-frequency characteristics. The details provide more detailed information about the morphology of the signal and reveal its high-frequency content. Information lost between two successive approximations is captured in the details part. From the second level of decomposition onwards, only the approximations obtained from the previous level are resubmitted for decomposition in a process similar to that explained below (Figure 3). The signal is decomposed into different frequency bands by means of successive high-pass and low-pass quadrature mirror filters, achieving a good time resolution at high frequencies and, at the same time, a good frequency resolution with long recordings.

**Figure 4:**
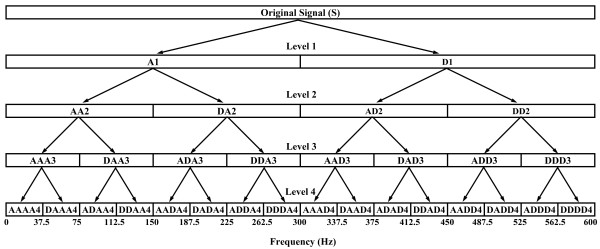
**Four-level wavelet packet decomposition tree**.

The wavelet packet analysis technique offers more detailed analysis than the DWT. In the DWT procedure, the generic step only splits the approximation portion (A_n_) into successive approximations (A_n+1_) and details (D_n+1_). The wavelet packet method extends this by also decomposing the detail part (D_n+1_) to obtain a complete decomposition tree. The decomposition tree employed is illustrated in Figure 4 using the appropriate MATLAB nomenclature [14]. The horizontal axis shows frequency range, i.e. zero to Nyquist frequency (600 Hz in this study). The signal is decomposed into 2^*j *^= 16 equally spaced frequency bands, *j *being the decomposition level (four in this study). For instance, wavelet packet analysis allows signal S to be represented as A_1 _+ AD_2 _+ ADD_3 _+ DDD_3_, providing an example of representation not possible with DWT.

**Figure 5:**
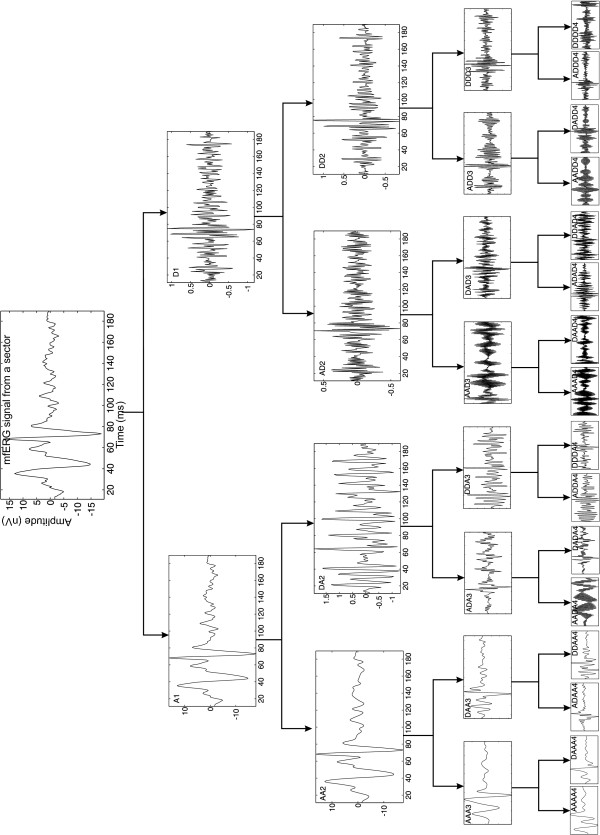
**Four-level wavelet packet decomposition of a control mfERG recording**.

**Figure 6:**
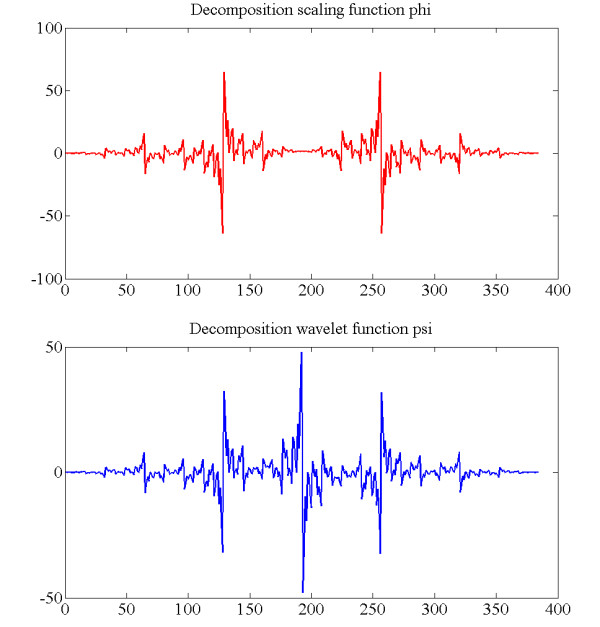
**The scaling function, Φ(t), and the wavelet function, Ψ(t), associated with Bior3.1. wavelet**.

The mfERG signals were analysed by applying up to four levels of wavelet packet decomposition to each of the different sectors for a time window of 10-190 ms. Figure 5 shows an example of wavelet packet decomposition of a control recording. This study analysed a great number of mother wavelets. Repeated testing showed that the Bior3.1 wavelet (Figure 6) performed best at identifying a marker in the signal reconstructed from the ADAA4 packet that could differentiate normal mfERG signals from those belonging to subjects with glaucoma.

**Figure 7:**
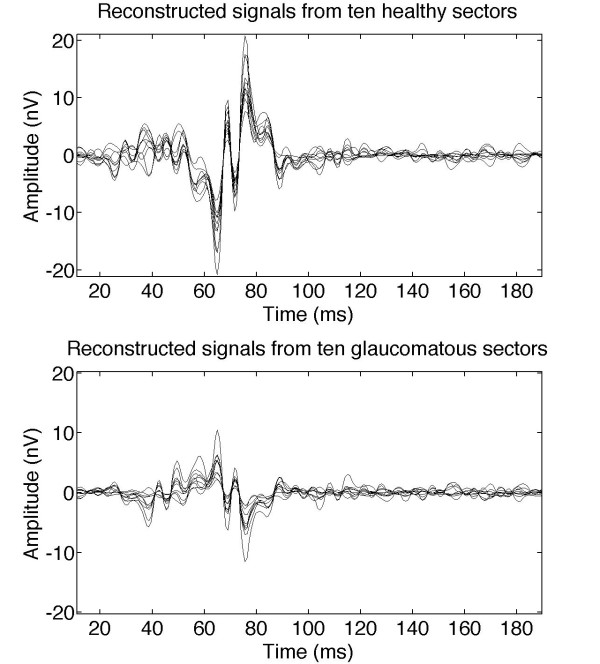
**Reconstruction of wavelet packet ADAA4**. Ten superimposed control recordings (upper graph) and ten glaucomatous recordings (lower graph) obtained from reconstruction of their ADAA4 wavelet packet.

After decomposition, inverse transformation was applied individually to all of the packets to convert them into a set of signals in the time domain. Several superimposed recordings were obtained from different sectors to gain an overview of which markers could differentiate normal signals from abnormal signals. Figure 7 represents 10 typical healthy records and 10 typical glaucomatous ones from 10 different controls and 10 different patients, respectively.

## Results

**Figure 8:**
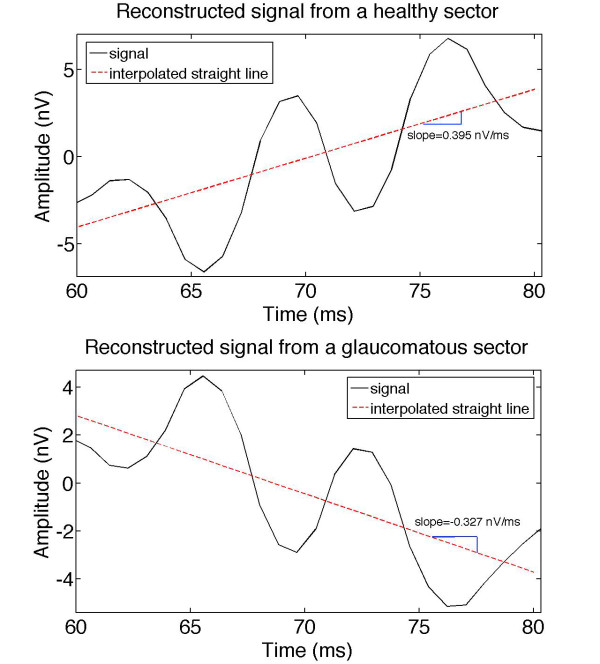
**Interpolated straight line from the 60-80 ms interval**. Signals resulting from reconstruction of wavelet packet ADAA4, corresponding to control (upper graph) and glaucomatous sectors (lower graph) from the analysis group.

Study of the analysis group revealed that each mfERG sector signal reconstructed from wavelet packet ADAA4 (the third packet in the fourth level of decomposition) showed a clear repetitive pattern in the time window running from 60-80 ms (see Figure 8). This consisted of a 1.5-cycle quasi-sinusoidal waveform section. The ADAA4 packet principally selects the frequency components of the recording between 75-112 Hz. In the case of the signals obtained from control mfERG recordings (Figure 8, upper graph), the sinusoidal waveform section shows a rising basal line (0.553 nV/ms ± 0.33 SD) that begins with a trough and ends with a peak. Conversely, the signals from glaucomatous mfERG recordings (Figure 8, lower graph), followed a falling basal line (-0.150 nV/ms ± 0.27 SD) (p < 0.01) and the sine wave is the inverse of the basal line for the normal control mfERG sectors.

**Figure 9:**
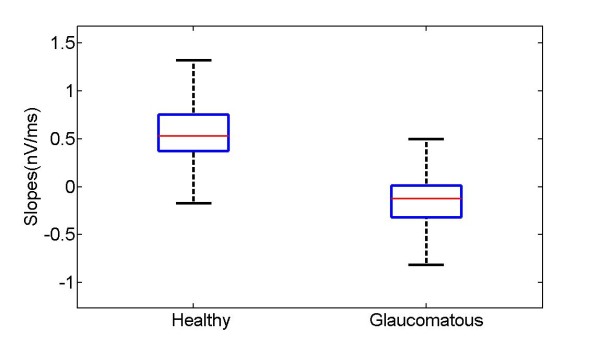
**Boxplot for the database of glaucomatous and control signals**.

The slope of the basal line was estimated by approximating the signal in the 60-80 ms time interval to a straight line using the method of least squares. Figure 9 shows the boxplot of slopes for the database of control and glaucomatous waveforms.

**Figure 10:**
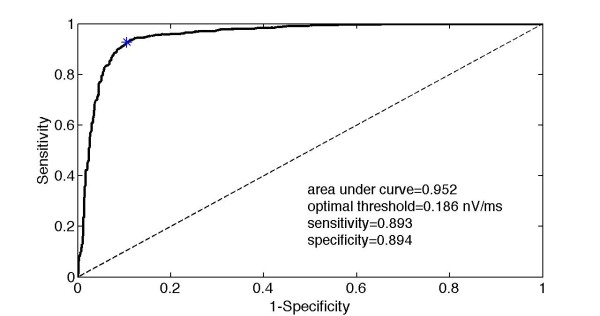
**ROC curve obtained by varying the threshold of the slope**.

A receiver operating characteristic (ROC) curve was then plotted using the analysis group mfERG recordings (Figure 10), modifying the value of the slope between the minimum (-1.130 nV/ms) and maximum (1.545 nV/ms) values. The threshold value that gave the nearest point to the (0, 1) vertex of the ROC diagram was accepted as the optimum decision threshold (t_opt_). A threshold of 0.186 nV/ms (t_opt_) on the slope provided the best differentiation between the normal and glaucomatous sectors. The value obtained for the area under the ROC curve was 0.952 and is a measure of the ability of the threshold to differentiate between control and glaucomatous sectors.

To validate wavelet packet analysis of the mfERG recording, it was tested on a group of five glaucomatous patients (one eye per patient, two left and three right eyes, 280 sectors in total). Table 1 shows the contingency table comparing the results obtained with wavelet packet analysis and the HVF diagnostic test. Application of the optimal threshold calculated in the validation cases showed that the technique proposed achieved a sensitivity of 0.81 and validation specificity of 0.73.

**Table 1:** Results obtained using the marker within the ADAA4 wavelet packet

mfERG (sectors)	Abnormal HVF (>10 dB)	Normal HVF (<10 dB)	
Abnormal mfERG-WP	60 sectors	55 sectors	PPV = 0.52
Normal mfERG-WP	14 sectors	151 sectors	NPV = 0.91
	Sensitivity = 0.81	Specificity = 0.73	

WP = wavelet packet, PPV = positive predictive value, NPV = negative predictive value. (p < 0.001, Fisher test)

## Conclusions

The global-flash mfERG paradigm protocol used in this study provides a reliable and objective measure of visual loss in glaucomatous patients. This stimulation paradigm was able to extract a large ONHC contribution from the mfERG responses, thereby making it easier to detect waveform abnormalities.

Analysis of the signals obtained from wavelet packet decomposition showed a clear repetitive pattern in the signal reconstructed from wavelet packet ADAA4 in the time interval running from 60-80 ms within the induced component. Applying the previously described analysis reveals a variation in the value of the slope of the basal line (Figure 8). In the case of recordings of normal sectors, the pattern consisted of a quasi-sinusoidal waveform section with a rising basal line, while recordings of glaucomatous sectors produced a falling basal line. The slope of the basal line was approximated by the method of least squares in the 60-80 ms time interval.

Application of the optimal threshold calculated in the validation cases showed good sensitivity and specificity. Nevertheless, a small percentage of sectors were still classified incorrectly (Table 1). In this respect, use of different types of amplitude and latency analysis on similar mfERG signals have also shown good sensitivity and specificity [15-17].

Studies of nerve fibre layer thickness have shown that glaucomatous damage can be present in the visual field hemifield with normal achromatic sensitivity [18]. In a recent study using FDP, it has been shown that in patients with OAG with established hemifield defects, 41% of 49 hemifields with apparently normal fields produced abnormal FDP results [19]. Also, several studies using SWAP show that this perimetric technique may be able to detect visual field defects before white-on-white perimetry in cases of suspected glaucoma and may detect earlier progression of visual field defects in glaucoma patients [20].

The principal purposes of this study were to develop a new mfERG-paradigm glaucoma analysis protocol and to gain a better understanding of how the mfERG and HVF techniques compare. However, this paper does not try to determine, at this stage, whether mfERG or automated achromatic perimetry is better at detecting glaucomatous damage. The authors are aware that such a longitudinal study would require a larger group of control subjects and patients tested with both techniques so that specificity, sensitivity and likelihood ratios could be correctly determined.

This study provides evidence that this new mfERG analysis method may be reliable enough to detect and map functional deficits that are not apparent using current automated perimetry tests. As new stimulation and analysis protocols develop, the authors believe that mfERG has the potential to become a useful tool in early detection of glaucoma-related functional deficits, as well as in longitudinal assessment of the same.

## Competing interests

The authors declare that they have no competing interests.

## Authors' contributions

JMM developed the study design, written the manuscript, carried out the evaluation study and analyzed the data. SO contributed to the evaluation tests design and was involved in the drafting and revision of the manuscript. LB and RB were involved in the drafting and revision of the manuscript. JMR contributed to the evaluation test design and performance. All authors read and approved the final version of the manuscript.
